# Ligand-dependent genomic function of glucocorticoid receptor in triple-negative breast cancer

**DOI:** 10.1038/ncomms9323

**Published:** 2015-09-16

**Authors:** Zhong Chen, Xun Lan, Dayong Wu, Benjamin Sunkel, Zhenqing Ye, Jiaoti Huang, Zhihua Liu, Steven K. Clinton, Victor X. Jin, Qianben Wang

**Affiliations:** 1 Department of Molecular Virology, Immunology and Medical Genetics, Comprehensive Cancer Center, Ohio State University College of Medicine, Columbus, Ohio 43210, USA; 2 Department of Genetics, Stanford University, Stanford, California 94305, USA; 3 Department of Molecular Medicine, University of Texas Health Science Center at San Antonio, San Antonio, Texas 78229, USA; 4 Departments of Pathology and Urology, Jonsson Comprehensive Cancer Center, David Geffen School of Medicine at UCLA, Los Angeles, California 90095, USA; 5 State Key Laboratory of Molecular Oncology, Cancer Institute and Hospital, Chinese Academy of Medical Sciences, Beijing 100021, China; 6 Division of Medical Oncology, Department of Internal Medicine, Ohio State University College of Medicine, Columbus, Ohio 43210, USA

## Abstract

Glucocorticoids (GCs) have been widely used as coadjuvants in the treatment of solid tumours, but GC treatment may be associated with poor pharmacotherapeutic response or prognosis. The genomic action of GC in these tumours is largely unknown. Here we find that dexamethasone (Dex, a synthetic GC)-regulated genes in triple-negative breast cancer (TNBC) cells are associated with drug resistance. Importantly, these GC-regulated genes are aberrantly expressed in TNBC patients and are associated with unfavourable clinical outcomes. Interestingly, in TNBC cells, Compound A (CpdA, a selective GR modulator) only regulates a small number of genes not involved in carcinogenesis and therapy resistance. Mechanistic studies using a ChIP-exo approach reveal that Dex- but not CpdA-liganded glucocorticoid receptor (GR) binds to a single glucocorticoid response element (GRE), which drives the expression of pro-tumorigenic genes. Our data suggest that development of safe coadjuvant therapy should consider the distinct genomic function between Dex- and CpdA-liganded GR.

## Abstract (web summary)

Glucocorticoids are widely used as coadjuvants in the treatment of solid tumours. Here, Chen *et al*. show that genes regulated by dexamethasone- but not Compound A-liganded glucocorticoid receptor are associated with therapy resistance and unfavourable clinical outcomes in triple-negative breast cancer.

## Graphical abstract

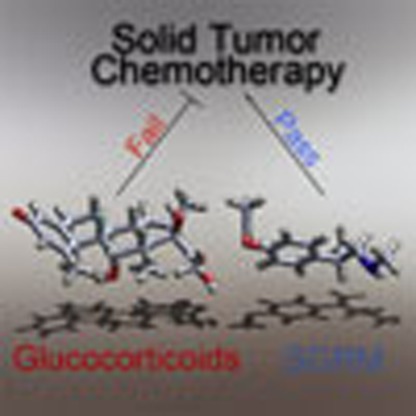

Glucocorticoids (GCs), functioning through the GC receptor (GR), play important roles in various physiological processes such as metabolism, immune response and development. Owing to their anti-inflammatory and immunosuppressive actions, GCs have been widely used in the treatment of inflammatory and autoimmune diseases^1^. In cancer therapies, GCs have not only been exploited in the treatment of lymphoid malignancies to induce cell apoptosis, but have also been used as co-medication with chemotherapy for solid tumours to reduce nausea and vomiting, inflammation as well as cytotoxic side effects^2^,^3^. Unfortunately, emerging evidence suggests that GCs and GC-activated GR may contribute to failure of chemotherapy and tumour progression of many types of solid tumours^4^,^5^. The underlying mechanisms of the pro-tumorigenic effects of GC-liganded GR in solid tumours are largely unknown.

The GC-liganded GR regulates target gene expression through binding to GC response elements (GREs), negative GRE (nGRE) or tethering to other transcription factors such as nuclear factor-κB (NF-κB) and AP1 (refs 1, 6). Unlike GC-liganded GR that either activates or represses downstream target genes, selective GR modulator (SGRM)-liganded GR negatively rather than positively regulates gene expression in most cases and this is believed to account for the retained anti-inflammatory activities and reduction of undesired effects of SGRMs^7^,^8^. For example, Compound A (CpdA), an SGRM that exerts potent anti-inflammatory activities by repressing proinflammatory genes, is unable to activate transcription of a few GRE-driven endogenous or reporter genes in model systems^9^,^10^. Thus, SGRMs such as CpdA may become alternative coadjuvants in the treatment of solid tumours. However, it is unknown whether SGRM- and GC-liganded GR globally regulate the same target genes in solid tumours. Addressing these issues is critical for developing and evaluating novel, safe coadjuvants for solid tumour therapy.

By comparing gene expression programmes regulated by dexamethasone (Dex)- and CpdA-liganded GR in triple-negative breast cancer (TNBC) cells and integrating our data sets with clinical breast cancer gene expression data sets, we find that Dex- but not CpdA-liganded GR binds to a precisely defined GRE, to directly regulate genes associated with breast cancer progression. These results elucidate the genomic mechanisms underlying the pro-tumorigenic effects of Dex-liganded GR in TNBC, and imply that CpdA may serve as a lead compound for developing safer coadjuvants for TNBC therapy.

## Results

### Association of *GR* expression with clinical outcomes

**Figure 1:**
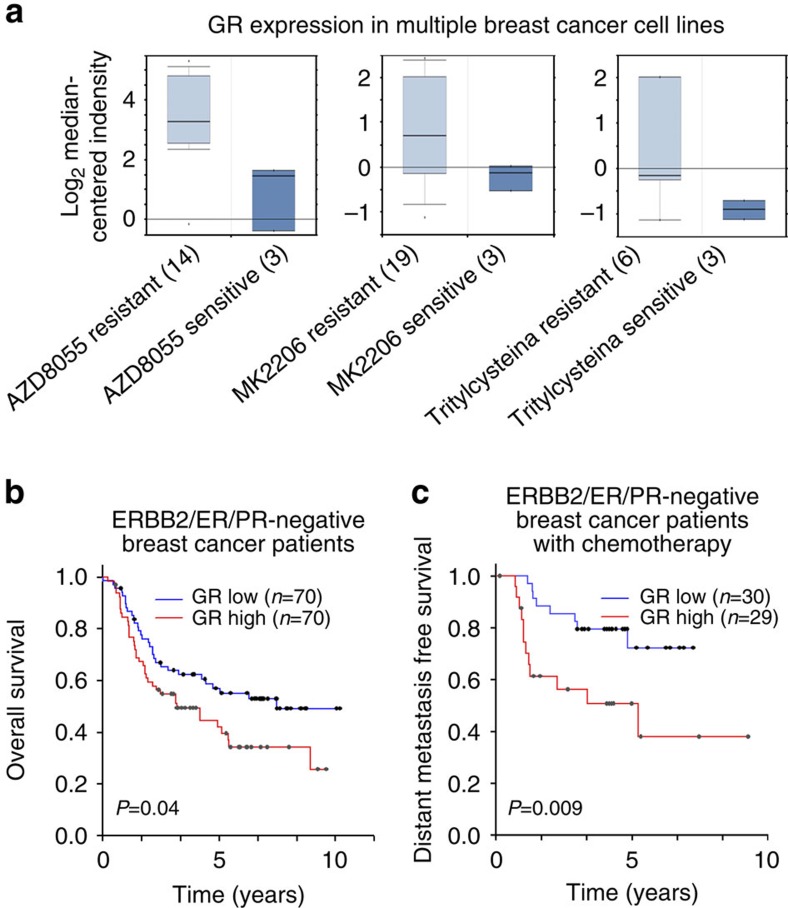
Correlation of *GR* expression with pharmacotherapy resistance of breast cancer cells and clinical outcomes of breast cancer patients. (**a**) Correlation of *GR* expression with resistance to the mammalian target of rapamycin inhibitor AZD8055, AKT inhibitor MK2206 and mitotic kinesin Eg5 inhibitor *S*-trityl-L-cysteine in multiple breast cancer cell lines. The numbers in the brackets indicate the numbers of different breast cancer cell lines. The gene expression data are from a previous study^12^. (**b**) Kaplan–Meier analysis comparing overall survival of a cohort of TNBC patients distinguished by low versus high expression of the *GR* gene. The clinical gene expression data are from a previous study^14^. (**c**) Kaplan–Meier curves comparing distant metastasis-free survival of another cohort of TNBC patients undergoing chemotherapy distinguished by low versus high expression of the *GR* gene. The clinical gene expression data are from a previous study^15^.

Analysis of *GR* gene expression revealed it to be ranked in the top 50% of genes expressed in 10 out of 16 solid tumours analysed (Supplementary Fig. 1) and this prompted us to study the potentially important role of GR in treatment response and/or solid tumorigenesis. In breast cancer, although previous studies have found that GCs induce chemotherapy resistance in some types of breast cancer cells^4^,^11^, it remains unclear whether resistance to chemotherapy or other pharmacotherapies is associated with *GR* expression. We therefore analysed gene expression profiles from pharmacotherapy-sensitive and -resistant breast cancer cell lines^12^ and found that high *GR* expression was correlated with resistance to the mammalian target of rapamycin inhibitor AZD8055, AKT inhibitor MK2206 and mitotic kinesin Eg5 inhibitor *S*-trityl-L-cysteine (Fig. 1a). As GR has extensive cross-talks with oestrogen receptor (ER) and progesterone receptor (PR) in breast cancer^6^,^13^, we next focused on studying the role of GR in ER/PR/HER2 TNBC, to highlight the direct effect of GR on breast carcinogenesis and therapy response. Analysis of *GR* expression in a cohort of TNBC patients^14^ found that high expression of *GR* was associated with shorter overall survival (Fig. 1b). In addition, high expression of *GR* was correlated with shorter metastasis-free survival in another cohort of TNBC patients undergoing chemotherapy^15^ (Fig. 1c). These results suggest that high GR expression/activation is correlated with poor therapeutic response and/or prognosis in TNBC. Importantly, analysis of *GR* expression in cohorts of patients with colorectal adenocarcinoma, superficial bladder cancer, ovarian serous cystadenocarcinoma and squamous cell lung carcinoma also revealed that increased *GR* expression was associated with poor prognosis (Supplementary Fig. 2). Together, these findings suggest that GR plays an important functional role in many types of solid tumours.

### Gene expression profiles induced by Dex and CpdA in TNBC

**Figure 2:**
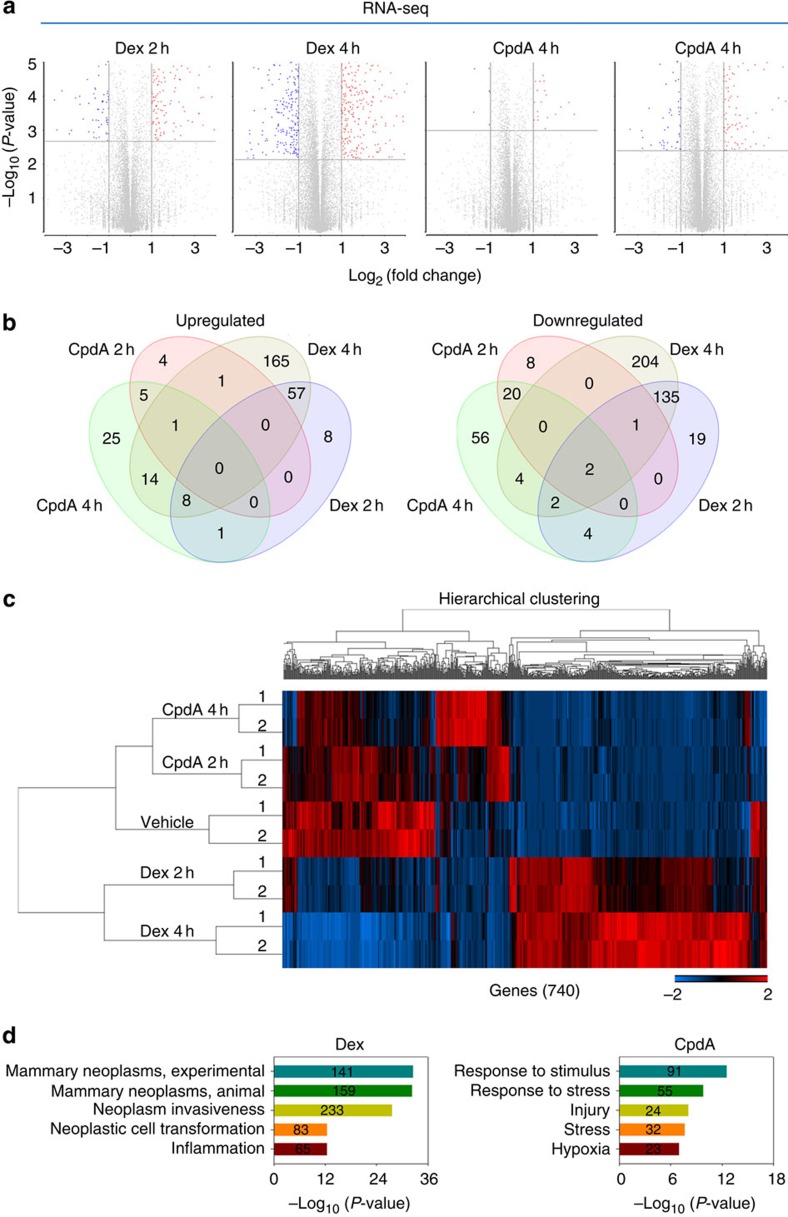
Analysis of Dex- and CpdA-stimulated transcriptomes in MDA-MB-231 cells. (**a**) Volcano plots of pairwise gene expression changes in response to 100 nM Dex for 2 and 4 h, or 10 μM CpdA for 2 and 4 h. Significant differentially expressed genes (fold>2) are highlighted in colour (red for upregulated genes and blue for downregulated genes) with FDR<0.05 (horizontal line). (**b**) Venn diagrams show upregulated (left panel) and downregulated (right panel) genes regulated by 2 and 4 h Dex or CpdA treatment. (**c**) A heatmap of differentially expressed genes after CpdA or Dex treatment at indicated time points. Genes varied across all samples after treatment with FDR<0.05 were used in hierarchical clustering. The gene expression (reads pe kilobase per million mapped reads (RPKM)) values for each gene were normalized to the standard normal distribution to generate *Z*-scores. The scale bar is shown with the minimum expression value for each gene in blue and the maximum value in red. (**d**) Enriched Gene Ontology (GO) terms in Dex- and CpdA-regulated genes.

We next investigated the mechanisms underlying the clinical relevance of GR in TNBC using MDA-MB-231 TNBC cells that harbour a mutant p53 (ref. 16), as our analysis revealed that TNBC patients exhibit a significantly higher frequency (36.3%) of p53 mutation than other breast cancer patients (9.2%) (Supplementary Fig. 3). To identify GR-regulated genes, we first performed an RNA-sequencing (RNA-seq) assay in MDA-MB-231 cells treated with vehicle or 100 nM Dex for 2 and 4 h. To ask whether CpdA regulates genes similar to or distinct from Dex, we also performed RNA-seq analysis in MDA-MB-231 cells stimulated with vehicle or 10 μM CpdA for 2 and 4 h. In two biological replicates with high reproducibility (Supplementary Fig. 4a), we found that Dex regulated a much larger number of genes than CpdA did at both time points, including both upregulated and downregulated genes (Fig. 2a,b and Supplementary Data 1). Importantly, unsupervised hierarchical clustering of gene expression data clearly distinguished Dex-regulated genes from CpdA-regulated genes, and CpdA- but not Dex-treated samples were clustered together with vehicle-treated samples based on their similarities in gene expression patterns (Fig. 2c and Supplementary Fig. 4b). Therefore, CpdA regulates a distinct class of genes from Dex in TNBC cells. Among genes regulated by Dex are those involved in mammary neoplasms and neoplastic cell transformation, whereas the small numbers of genes regulated by CpdA are not associated with cancer-related processes (Fig. 2d). Together, these data suggest that Dex but not CpdA regulates a gene expression programme involved in the carcinogenesis of TNBC.

### Dex regulates genes involved in TNBC progression

**Figure 3:**
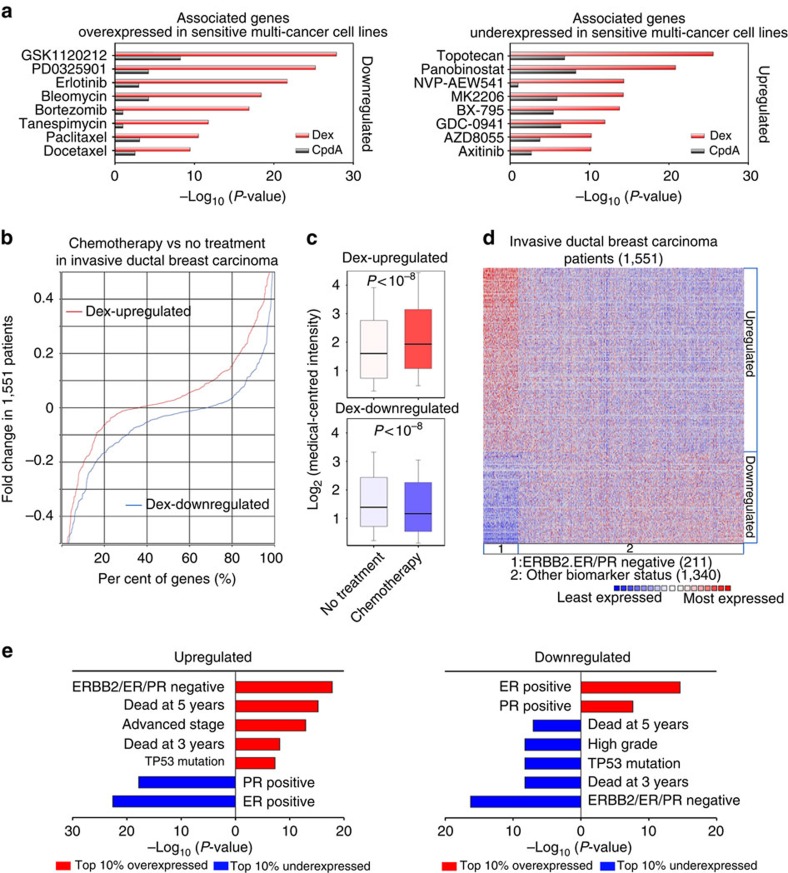
Functional and clinical association analysis of Dex- and CpdA-regulated genes. (**a**) Correlation of Dex- and CpdA-regulated genes with genes differentially expressed between drug-sensitive and -resistant multiple cancer cell lines. (**b**) Cumulative distribution of ratios of gene expression change in patients receiving chemotherapy (359 patients) relative to those not receiving chemotherapy (202 patients) were plotted using Dex-upregulated genes and Dex-downregulated genes in MDA-MB-231 cells. (**c**) Box plots show changes in expression of Dex-upregulated genes (upper panel) and Dex-downregulated genes (lower panel) in patients receiving chemotherapy versus those not receiving chemotherapy. (**d**) Genes upregulated and downregulated by both Dex treatment in TNBC cells and chemotherapy in patients are overexpressed and underexpressed, respectively, in invasive ductal TNBC (211 patients) but not in other breast cancer subtypes (1,340 patients). (**e**) Clinical association analysis was performed using upregulated and downregulated genes from **d** and the top 10% over/underexpressed genes in invasive ductal breast carcinoma patients.

To assess the functional and clinical significance of Dex- and CpdA-regulated genes, we first correlated Dex- and CpdA-regulated genes with genes differentially expressed between multiple drug-sensitive/resistant cancer cell lines^12^,^17^. We observed a much stronger association between genes that were underexpressed in drug-resistant cell lines and Dex- versus CpdA-downregulated genes (Fig. 3a). Similarly, compared with CpdA-upregulated genes, Dex-upregulated genes had markedly higher correlation with overexpressed genes in drug-resistant cells (Fig. 3a). These data suggest that Dex- but not CpdA-regulated genes are highly associated with drug resistance in cancer cells. As Dex is frequently administered during chemotherapy for solid tumours to reduce side effects^2^,^3^, we next correlated Dex-regulated genes with clinical breast cancer by analysis of gene expression data sets from a large cohort of 1,551 patients with invasive ductal breast carcinoma^14^. This revealed a striking association between Dex-upregulated genes in TNBC cells and upregulated genes in patients receiving chemotherapy versus those not receiving chemotherapy (Fig. 3b,c). Similarly strong correlation was observed between Dex-downregulated genes in TNBC cells and downregulated genes in patients receiving chemotherapy (Fig. 3b,c). Importantly, genes that were upregulated or downregulated by Dex treatment in TNBC cells, and which showed corresponding overexpression or underexpression in patients receiving chemotherapy, were also overexpressed or underexpressed in TNBC patients compared with patients with other breast cancer subtypes, respectively (Fig. 3d and Supplementary Data 2). These differentially regulated genes were significantly associated with status of ER, PR and ERBB2, tumour grade and stage, p53 mutation and clinical outcomes of breast cancer patients^14^ (Fig. 3e). Collectively, these data suggest that Dex-regulated genes are associated with breast cancer progression.

### Ligand-dependent genomic binding of GR in TNBC

**Figure 4:**
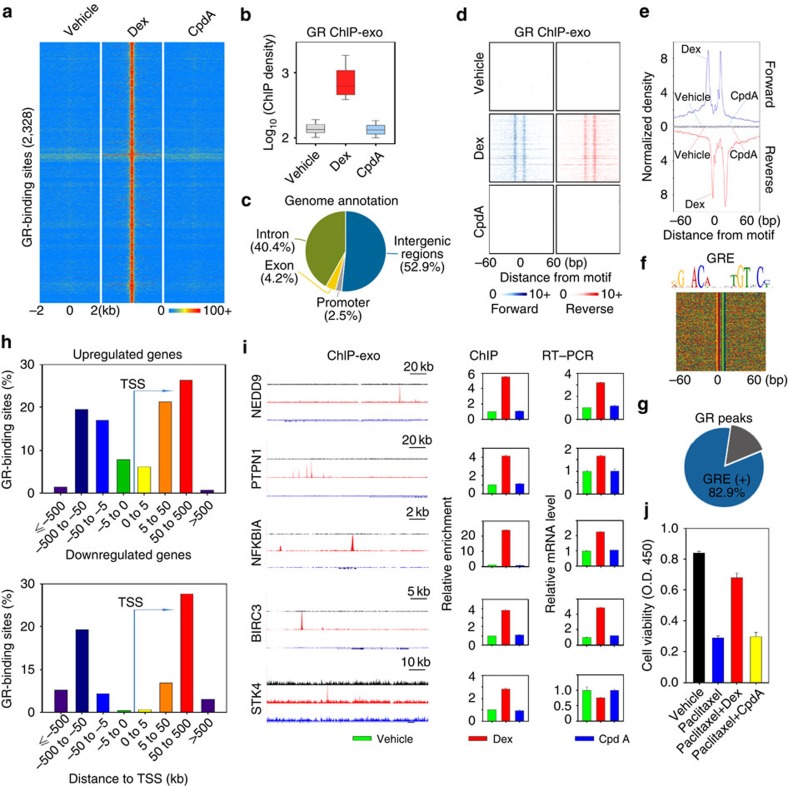
Precise definition and characterization of the GRE recognized by Dex-liganded GR in MDA-MB-231 cells. (**a**) Heat maps show the signal intensity of GR binding in MDA-MB-231 cells treated with vehicle, 100 nM Dex or 10 μM CpdA for 1 h. The number (2,328) indicates Dex-responsive GR locations. (**b**) A box plot shows ChIP-exo signal densities in Dex-responsive GR locations in cells treated with vehicle, Dex or CpdA. (**c**) Classification of specific Dex-responsive GR-binding locations based on annotation. (**d**) Raw tags distribution and (**e**) aggregated tag density over Dex GRE under different treatment conditions is shown on the forward (blue) and reverse (red) strands, separately. (**f**) Dex GRE is shown and the sequences are presented in the same order as in **d**. (**g**) Percentage of Dex-responsive GR-binding locations containing Dex GRE. (**h**) The distribution of Dex GR binding around the transcription start site (TSS) of upregulated (upper panel) and downregulated (lower panel) GR target genes are shown. (**i**) Left panel: UCSC genome browser views of ChIP-exo sequencing data at five gene loci. Y-scale is the same for each gene locus. Colours represent different treatment conditions: vehicle (green), red (Dex) and blue (CpdA). Middle panel: ChIP validation of GR ChIP-exo data. Cells were treated with vehicle, 100 nM Dex or 10 μM CpdA for 1 h and GR ChIP was performed. For the *PTPN1* locus, ChIP was performed to validate the binding of GR to the highest binding peak. Right panel: messenger RNA levels of five genes were also examined by reverse transcriptase–PCR (RT–PCR) assays. ChIP and RT–PCR data are the mean of triplicates±s.d. (**j**) Cells were treated with vehicle, 100 nM paclitaxel, 100 nM paclitaxel/100 nM Dex or 100 nM paclitaxel/10 μM CpdA and cell proliferation was determined on day 4 using the WST-1 assay. The data are the mean of triplicates±s.d.

To elucidate the molecular mechanisms underlying the differential gene expression between Dex- and CpdA-treated TNBC cells, we mapped GR-binding regions and precisely defined GR-binding motifs in drug-treated MDA-MB-231 cells using our modified chromatin immunoprecipitation-exonuclease (ChIP-exo) method with the newly developed ChIP-exo algorithm^18^. Cells were treated with vehicle, 100 nM Dex or 10 μM CpdA for 1 h and GR ChIP-exo assays were performed. Using the BELT algorithm^19^, we identified 2,328 Dex-responsive GR-binding locations and 819 CpdA-responsive GR-binding locations (Fig. 4a,b and Supplementary Fig. 5a–c). There was little overlap between these two sets of GR-binding locations (Supplementary Fig. 5a). The vast majority of Dex- and CpdA-responsive GR-binding locations were located at non-promoter regions (Fig. 4c and Supplementary Fig. 5d).

Using a border pattern-based motif defining approach (BPMotif)^18^, we discovered a GRE with clear borders (that is, exonuclease stop sites) enriched within Dex-responsive GR-binding regions (Fig. 4d–f). Strikingly, 82.9% of Dex-responsive GR-binding locations had this precisely defined GRE (Fig. 4g). Although the AP1 motif was also enriched in Dex-responsive GR locations, its frequency is very low (Supplementary Fig. 6a). More importantly, no border signals were observed over the AP1 motif in GR ChIP-exo data (Supplementary Fig. 6b). We next performed AP1 ChIP-sequencing (ChIP-seq) in MDA-MB-231 cells treated with vehicle, 100 nM Dex or 10 μM CpdA for 1 h. Although we found that 35.3% of Dex-responsive regions overlapped with AP1-binding regions (Supplementary Fig. 6c), Dex treatment had no effect on AP1 binding to either AP1/GR-shared regions or AP1-specific binding regions (Supplementary Fig. 7a–d). These results suggest that AP1 may function primarily by enhancing chromatin accessibility^20^ to facilitate Dex-liganded GR binding rather than by tethering GR to chromatin. In addition, neither the NF-κB motif nor nGREs were enriched/protected from exonuclease digestion (Supplementary Fig. 6d,f) and Dex-responsive GR-binding locations show very little overlap with NF-κB-binding sites (Supplementary Fig. 6e) discovered by NF-κB ChIP-seq (Supplementary Fig. 7e–h). Taken together, these data suggest that direct binding of GR to the precisely defined GRE is the predominant mechanism for Dex-stimulated GR binding in TNBC cells. To identify target genes regulated by Dex-responsive GR-binding locations, we used an integrated approach combining the Genomic Regions Enrichment of Annotations Tool (GREAT) algorithm^21^ and our RNA-seq data (Fig. 2). Although most GR binding locations were found at non-promoter regions around Dex-regulated genes, GR bound closer to upregulated genes than to downregulated genes (Fig. 4h). These results suggest that GR/GRE interaction drives the expression of Dex-regulated genes. Interestingly, genes near CpdA-responsive GR-binding locations identified by GREAT show very little overlap with CpdA-regulated genes determined by RNA-seq (Supplementary Fig. 5e,f), suggesting that CpdA-liganded GR may not directly regulate most, if not all, CpdA-responsive genes in TNBC cells. To ask how CpdA-liganded GR interacts with chromatin, we performed motif analysis in CpdA-responsive GR-binding locations using BPMotif. Interestingly, the most significantly enriched motif with border signals was a novel motif (YCTYCCH) distinct from the GRE (Supplementary Fig. 5g,h). The specific interactions between GR and this motif was confirmed *in vitro* using electrophoretic mobility shift assay (EMSA) (Supplementary Fig. 5i,j). In addition, although previous studies showed that CpdA inhibited DNA binding and transactivation of GR-tethering factors such as AP1 and NF-κB *in vitro*^9^,^10^,^22^,^23^, neither NF-κB nor AP1 motif was enriched in CpdA-responsive GR binding locations. Furthermore, CpdA treatment had little effect on AP1 or NF-κB binding (Supplementary Fig. 7a,b,e,f). These data suggest that CpdA-liganded GR does not bind to chromatin by tethering to AP1 or NF-κB in TNBC cells. Finally, motif analysis found almost no nGRE occurring in CpdA-responsive GR-binding locations, suggesting CpdA-liganded GR does not bind to nGRE. Collectively, these data suggest that although CpdA-liganded GR is able to directly bind to chromatin, other unknown mechanisms may be involved in CpdA-regulated gene expression in TNBC cells.

We next validated ChIP-exo and RNA-seq data by selecting the *BIRC3*, *PTPN1*, *NFκBIA*, *NEDD9* and *STK4* genes, as previous studies have found that these genes are involved in survival, proliferation, epithelial–mesenchymal transition, invasion or metastasis of TNBC and other cancers^11^,^24^,^25^,^26^,^27^,^28^. Validation of ChIP-exo results by standard ChIP assays found that Dex induced GR binding to GREs in the regulatory regions of these genes, whereas CpdA failed to stimulate GR recruitment to these regions (Fig. 4i). Although Dex stimulation increased expression of *BIRC3*, *PTP1B*, *NFκBIA* and *NEDD9* that function as oncogenes^11^,^24^,^26^,^28^, it decreased expression of *STK4* that acts as a tumour suppressor^25^,^27^ (Fig. 4i). Importantly, consistent with the failure of CpdA to induce GR binding to regulatory regions of these genes, CpdA treatment had no effect on the expression of these genes (Fig. 4i). Together, these data clearly demonstrate that CpdA-liganded GR does not bind to GREs and thus fails to regulate GRE-driven genes involved in breast tumorigenesis. To begin to evaluate the translational significance of our genomic findings, we investigated whether Dex and CpdA play differential roles in chemotherapeutic growth inhibition. We measured proliferation in cells treated with vehicle, a chemotherapy drug paclitaxel, paclitaxel combined with Dex or paclitaxel combined with CpdA. Although Dex markedly protected TNBC cells from growth inhibition, CpdA has no such effects (Fig. 4j). These data suggest that CpdA may be safer than Dex when co-administrated with chemotherapy in TNBC patients.

## Discussion

By using an integrated approach combing GR ChIP-exo, AP1/NF-κB ChIP-seq and RNA-seq analysis in TNBC cells with analysis of multiple clinical breast cancer data sets comprising over 2,000 patients, we found that Dex-liganded GR binds to a precisely defined GRE, to directly regulate genes associated with drug resistance and unfavourable clinical characteristics and outcomes (Figs 2, 3, 4). Although a recent GR ChIP-exo study found that Dex-liganded GR binds to highly degenerate sequences in other cell models^29^, we found that 82.9% of Dex-responsive GR-binding locations contain this single GRE with very clear border signals in TNBC cells (Fig. 4d–g). Consistent with a recent report demonstrating that GR transcriptional outcomes are not determined by GRE types^30^, we show that Dex-liganded GR binds to the precisely defined GRE to both activate oncogenes (for example, *BIRC3* and *NEDD9*) and repress tumour suppressor genes (for example, *STK4*) in TNBC cells (Figs 2, 3, 4). Surprisingly, our ChIP-exo analysis in TNBC cells did not reveal that GR binds on chromatin to the putative nGRE motif identified by motif scanning^31^ (Supplementary Fig. 6f). Consistent with our findings, the two most recent GR ChIP-exo studies in other cells/tissues (for example, IMR90 cells and mouse liver) also found that Dex-liganded GR does not bind to nGREs^29^,^32^. These data suggest that the prevalence of the nGRE in the genome and the extent to which this motif is used by GR need to be scrutinized.

Although co-treatment with GCs such as Dex in many types of solid tumours is effective in easing symptoms related to chemotherapy or cancer *per se*^2^,^3^, GC therapy may increase the risk for failure of chemotherapy^3^,^4^,^5^,^11^. CpdA has been reported to exert potent anti-inflammatory effects in various experimental disease models without undesired effects such as inducing hyperglycaemia, suppressing the hypothalamic–pituitary–adrenal axis or impairing intestinal epithelial cell restitution^8^,^9^,^22^,^33^. In this study, we found that CpdA-liganded GR does not bind to genomic regions occupied by Dex-liganded GR (Fig. 4a,b and Supplementary Fig. 5a) and thus fail to regulate Dex-regulated genes involved in drug resistance and breast carcinogenensis (Figs 2, 3, 4). Although CpdA induces distinct conformational changes of GR^8^,^9^ that may allow CpdA-liganded GR to recognize a distinct GR motif on chromatin (Supplementary Fig. 5g–j), genes associated with CpdA-responsive GR binding are distinct from the small number of CpdA-regulated genes identified by RNA-seq (Supplementary Fig. 5e,f). It is possible that CpdA-liganded GR-associated genes are weakly responsive to short-term CpdA stimulation and/or can be strongly regulated following long-term CpdA treatment. As suggested by previous studies^7^, CpdA may also function through another receptor or factor. Nevertheless, those CpdA-regulated genes are not associated with cancer-relevant processes and drug resistance (Figs 2d and 3a). These data suggest that CpdA may be a safer coadjuvant for chemotherapy. Indeed, our studies found that Dex but not CpdA treatment markedly protects TNBC cells from chemotherapy-induced growth inhibition (Fig. 4j). Future studies are required to assess the impact of CpdA or its derivatives in combination with chemotherapy in rodent models of TNBC, as well as other solid tumours. Future studies should also investigate how CpdA or its derivatives exerts anti-inflammatory activities in TNBC and other solid tumours.

## Methods

### Cell culture and antibodies

The TNBC cell line MDA-MB-231 was obtained from the American Type Culture Collection and cultured in DMEM complete medium. For hormone-responsive experiments, MDA-MB-231 cells were maintained in phenol red-free medium with 5% charcoal-stripped fetal bovine serum for 3 days and then treated with vehicle and different ligands. Antibodies used were anti-GR (E-20), anti-c-Jun (H-79) and anti-NFκB p65 (C-20) from Santa Cruz Biotechnology (Santa Cruz, CA) and anti-GR (3660) from Cell Signaling Technology (Billerica, MA).

### RNA-seq and data analysis

MDA-MB-231 cells were treated with 100 nM Dex or 10 μM CpdA for 2 and 4 h, respectively. RNA was extracted using the RNeasy Mini Kit (Qiagen, Valencia, CA). Complementary DNA libraries were constructed using the Illumina Truseq RNA Sample Prep Kit according to the manufacturer’s protocol. Fifty base pairs of single-end reads were generated on the Illumina HiSeq 2500 platform at the Ohio State University Comprehensive Cancer Center (OSUCCC) sequencing core with three multiplexed samples per lane. Read alignment was conducted using TopHat 2.0.13, and relative transcript abundances and differentially expressed genes were determined using Partek Genomics Suite (v6.6) with default settings. All biological duplicates show high correlation around 0.99 (Spearman). Hierarchical clustering was performed using genes with a false discovery rate (FDR)<0.05 and a fold change of 2 (Fig. 2) or 1.5 (Supplementary Fig. 4). For Gene Ontology analysis of Dex- or CpdA-regulated genes (fold change>2 and FDR<0.05), the top five Biological Processes (Gene Ontology) or Diseases (MeSH) were selected based on the statistical significance for each category using Genomatix Pathway System (v3.3).

### Survival analysis

Patients were stratified according to expression of GR signature in different data sets and the top one-third or -fourth and bottom one-third or -fourth of patients were compared. Overall survival was calculated from sampling date to date of death or last follow-up. Kaplan–Meier survival curves were generated and compared using the log-rank test with SigmaPlot (13.0).

### Meta-analysis

The meta-analysis of global gene expression data was performed in the Oncomine database. Gene expression profiles of multiple data sets were integrated. Genes scoring in the top 5 or 10% of outliers were selected on the basis of the median *P*-value of the median gene rank in overexpression or underexpression percentiles across the data sets.

### ChIP-exo and data analysis

ChIP-exo was performed and analysed as previously described^18^. Briefly, MDA-MB-231 cells were treated with vehicle, 100 nM Dex or 10 μM CpdA for 1 h. After fixation with 1% formaldehyde for 10 min at room temperature, chromatin was sonicated and incubated with 4 μg GR antibody overnight. T4 DNA polymerase, T4 PNK and Klenow DNA Polymerase were used together for end polishing. The ligation step was performed with 1 mM dithiothreitol. Protein A Dynal magnetic beads were washed using modified RIPA buffer (50 mM Tris-HCl pH 7.8, 1 mM EDTA, 0.25% Na deoxycholate, 1% NP-40, 0.5 M LiCl) followed by Tris pH 8.0 twice during each step. The library was amplified with only ten cycles and prepared without gel-based size selection. Paired-end sequencing (50 bp) was performed using Illumina HiSeq 2000 at the OSUCCC sequencing core (Supplementary Table 1). Raw reads were aligned to the human reference genome (hg19) using bowtie with default parameter settings. Clonal reads and bad quality reads were removed. All biological duplicates show high Spearman’s rank correlation (*r*_Vehicle_=0.982, *r*_Dex_=0.959 and *r*_CpdA_=0.983).

The enriched DNA motifs were defined by a multi-phase cross-validation procedure. Genomics Suite *v*6.6 (Partek) and MEME Suite *v*4.9 (ref. 34) were used to find the candidate motifs. Initial motif candidates were generated using default programme settings (one instance per sequence, <40 bp of border extension). Motifs were then clustered with the Pearson’s correlation coefficient. Exo signal was measured to define border patterns and classify motifs. A set of overrepresented motifs was then used to correct border extension according to the enriched motif position. Motif discovery was repeated twice. Motifs with *E* <1e−100 or that were found in 10% of sequences were retained as reliable predictions for the next round of analysis. Finally, we identified motifs satisfying the following extensible criteria: (1) motif similarity compared with GRE in the TF-binding databases or between core motifs defined in GR ChIP-exo data; (2) at least one common protected border exists upstream and downstream of the strand-specific motif; and (3) same distance from borders to the most conserved nucleotides in variable motifs. For those core motifs that did not meet criteria (1), we also performed motif comparison and clustering based on criteria (2) and (3) using the exo-defined matrix.

### ChIP-seq and data analysis

ChIP-seq was performed as previously described^18^. Briefly, cells were grown to 70%–80% confluence and were cross-linked with 1% formaldehyde for 10 min at room temperature. After washing twice with cold PBS, cells were collected and resuspended in lysis buffer (1% SDS, 5 mM EDTA, 50 mM Tris pH 8.0, 1 × protease and phosphatase inhibitors). After sonication, the soluble chromatin was diluted in 1% Triton X-100, 2 mM EDTA, 150 mM NaCl, 20 mM Tris pH 8.0, 1 × protease and phosphatase inhibitors and incubated with 4 μg of antibodies overnight. The eluted ChIP DNA was used for library generation with NEBNext ChIP-Seq Library Prep Master Mix Set according to the manufacturer’s protocol. The library was amplified with 12 PCR cycles and prepared with gel-based size selection (250 bp). The sequencing was performed using Illumina HiSeq 2500 at the OSUCCC sequencing core. Raw reads were aligned to the human reference genome (hg19) using bowtie with default parameter settings. Clonal reads and bad quality reads were removed. The tracks of coverage density were made with extended reads, which have been normalized to the same sequencing depth (100 million). Uniquely mapped reads were used for peak calling using BELT^19^ with FDR<0.005.

### Binding-gene correlation

GREAT^21^ was used to predict functions of GR-binding sites and identify all genes around binding sites in MDA-MB-231 cells. Resulting gene lists were filtered based on differentially expressed genes determined by RNA-Seq ([Fold Change]>1.2, *P*-value≤0.05, FDR≤0.05).

### Differential enrichment analysis

The BELT programme^19^ was used to identify different ligand-stimulated locations in MDA-MB-231 cells. BELT compared the read densities of enriched locations in Dex/vehicle and CpdA/vehicle using Fisher’s exact test, with a *P*-value cutoff of 0.05.

### Real-time reverse transcriptase–PCR and ChIP

For real-time reverse transcriptase–PCR^35^, total RNA was isolated using an RNeasy kit (Qiagen). cDNA was reverse transcribed from total RNA (2 μg) using a High Capacity cDNA Reverse Transcription Kit (Applied Biosystems, Foster City, CA). Real-time PCR analysis was performed using Power SYBRs Green PCR Master Mix (Applied Biosystems) on the StepOnePlusTM Real-Time PCR System (Applied Biosystems) following the manufacturer’s instructions. For ChIP assay^35^, chromatin was cross-linked for 10 min at room temperature with 1% formaldehyde. The chromatin was immunoprecipitated with 4 μg of antibodies at 4 °C overnight. The reverse cross-linked ChIP DNA was purified and then analysed by real-time PCR. The primers are listed in Supplementary Table 2.

### Electrophoretic mobility shift assay

EMSA probes were synthesized by Integrated DNA Technologies. The Gelshift Chemiluminescent EMSA kit (Active Motif, Carlsbad, CA) was used according to the manufacturer’s protocols. Baculovirus recombinant human GR protein (ab3582; 3.5 μg) for each condition were incubated with probes (20 fmol) (Supplementary Table 3) for 20 min on ice. For supershift, anti-GR (ab3578) from Abcam (Cambridge, MA) was incubated for 30 min after adding probes. The 5% TBE gel was run at 100 V for 1 h and then transferred to nylon membrane. After incubation with streptavidin–horseradish peroxidase conjugate, the membrane was exposed to film and developed.

### Cell proliferation assay

MDA-MB-231 cells were treated with vehicle, 100 nM paclitaxel, 100 nM paclitaxel/100 nM Dex or 100 nM paclitaxel/10 μM CpdA and cell proliferation was determined on day 4 using the WST-1 assay.

## Additional information

**Accession codes:** The ChIP-exo, ChIP-seq and RNA-seq data has been deposited in the GEO database under the accession code GSE56022.

**How to cite this article:** Chen, Z. *et al*. Ligand-dependent genomic function of glucocorticoid receptor in triple-negative breast cancer. *Nat. Commun.* 6:8323 doi: 10.1038/ncomms9323 (2015).

## Supplementary Material

Supplementary Information Supplementary Figures 1-7, Supplementary Tables 1-3 and Supplementary References

Supplementary Data 1 Genes differentially expressed by Dex and CpdA treatment in TNBC cells

Supplementary Data 2 Genes regulated by both Dex treatment in TNBC cells and chemotherapy in breast cancer patients
