# Supplementary material for: Ligand-dependent genomic function of glucocorticoid receptor in triple-negative breast cancer
Source: Nat Commun. 2015 Sep 16;6:8323. doi: 10.1038/ncomms9323 (PMC4573460; doi:10.1038/ncomms9323)
Supplement: Supplementary Information — Supplementary Figures 1-7, Supplementary Tables 1-3 and Supplementary References [file ncomms9323-s1.pdf]

Supplementary Figures

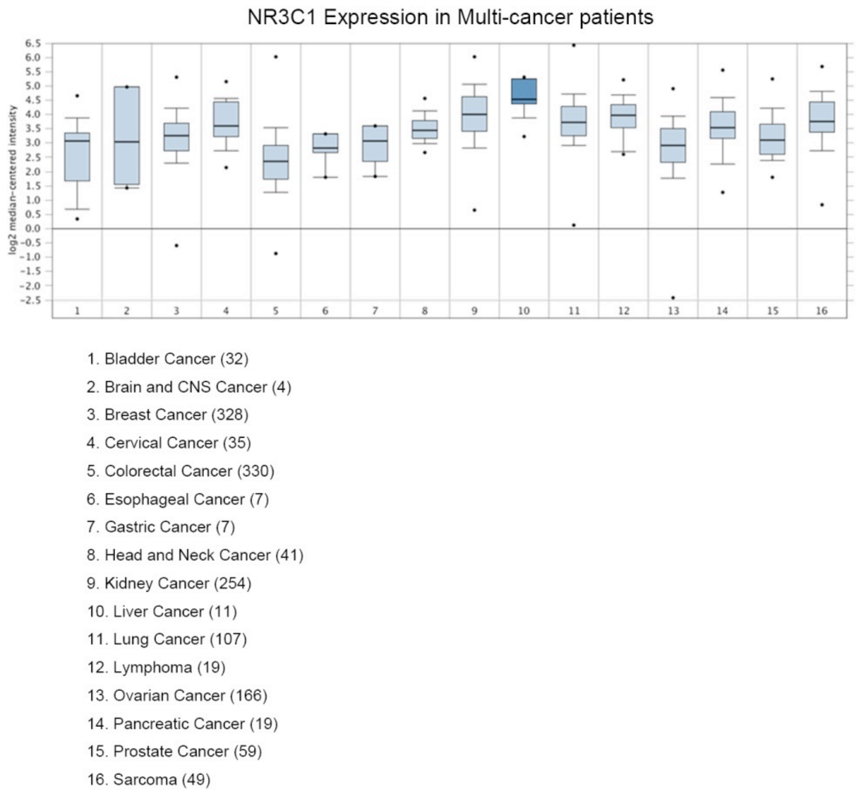

Supplementary Figure 1. Analysis of *GR* (*NR3C1*) gene expression in 16 types of tumors. Oncomine analysis[1] of Bittner Multi-cancer dataset showed that *GR* gene was expressed in the top 50% of highly expressed genes in 10 out of 16 types of solid tumors.

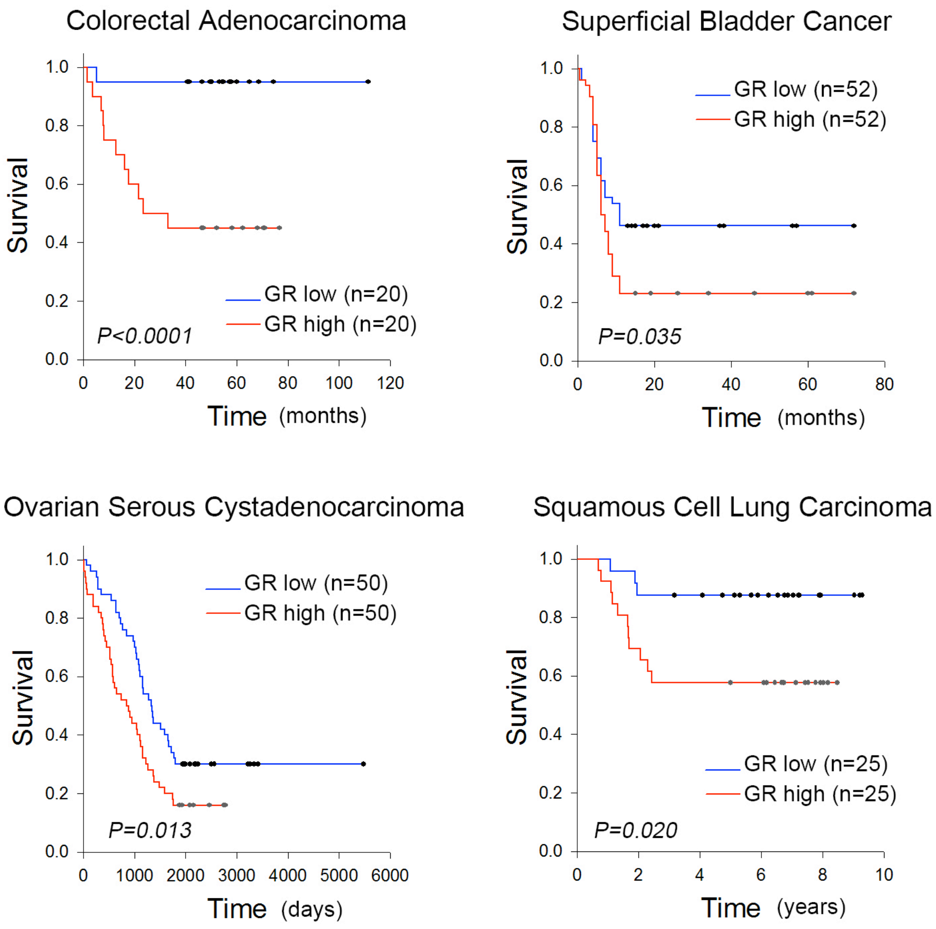

Supplementary Figure 2. Kaplan-Meier analysis comparing overall survival of patients with solid tumors distinguished by low versus high expression of the *GR* gene. The datasets used in survival analysis are all from Oncomine database[1].

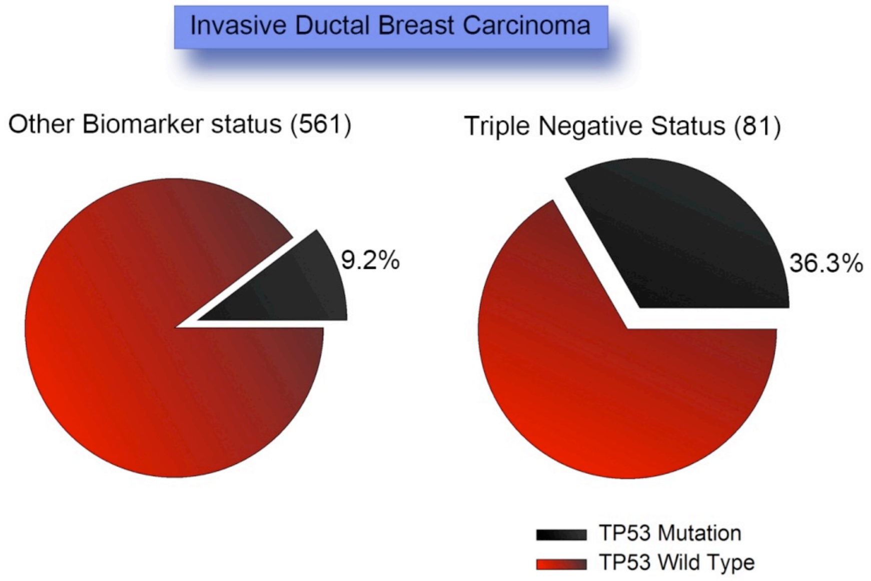

Supplementary Figure 3. Analysis of p53 mutation status in 81 TNBC patients and 561 patients with other breast cancer subtypes (patients data are from Curtis *et al*. dataset[2])**.**

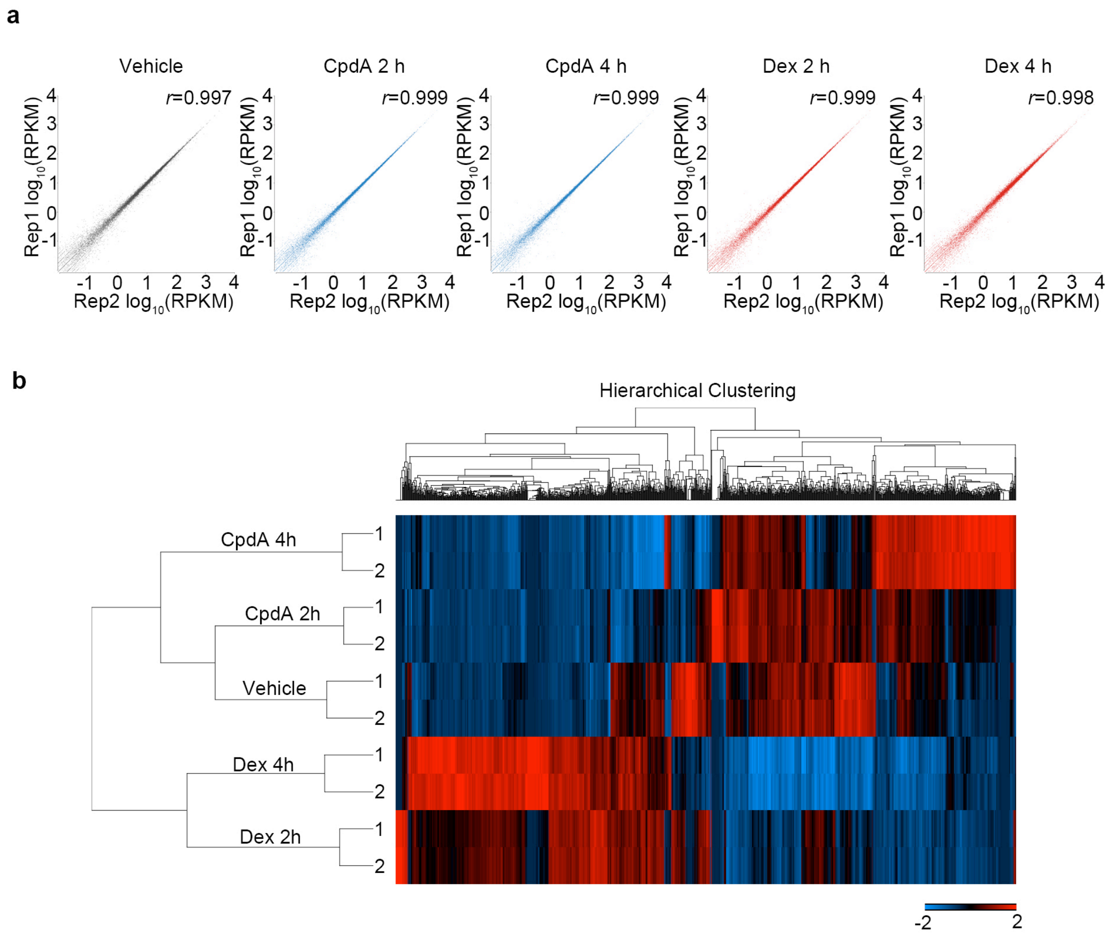

Supplementary Figure 4. RNA-seq analysis of MDA-MB-231 cells treated with Dex or CpdA. (a) Correlation between biological replicates of RNA-seq in cells treated with vehicle, 10 μM CpdA for 2 h and 4 h, or 100 nM Dex for 2 h and 4 h. **(b)** Cluster analysis of genes differentially expressed (Fold >1.5) by CpdA or Dex treatment. Unsupervised hierarchical clustering of genes (columns) from cells in different conditions (rows) was performed. Red and blue color represents upregulation and downregulation, respectively.

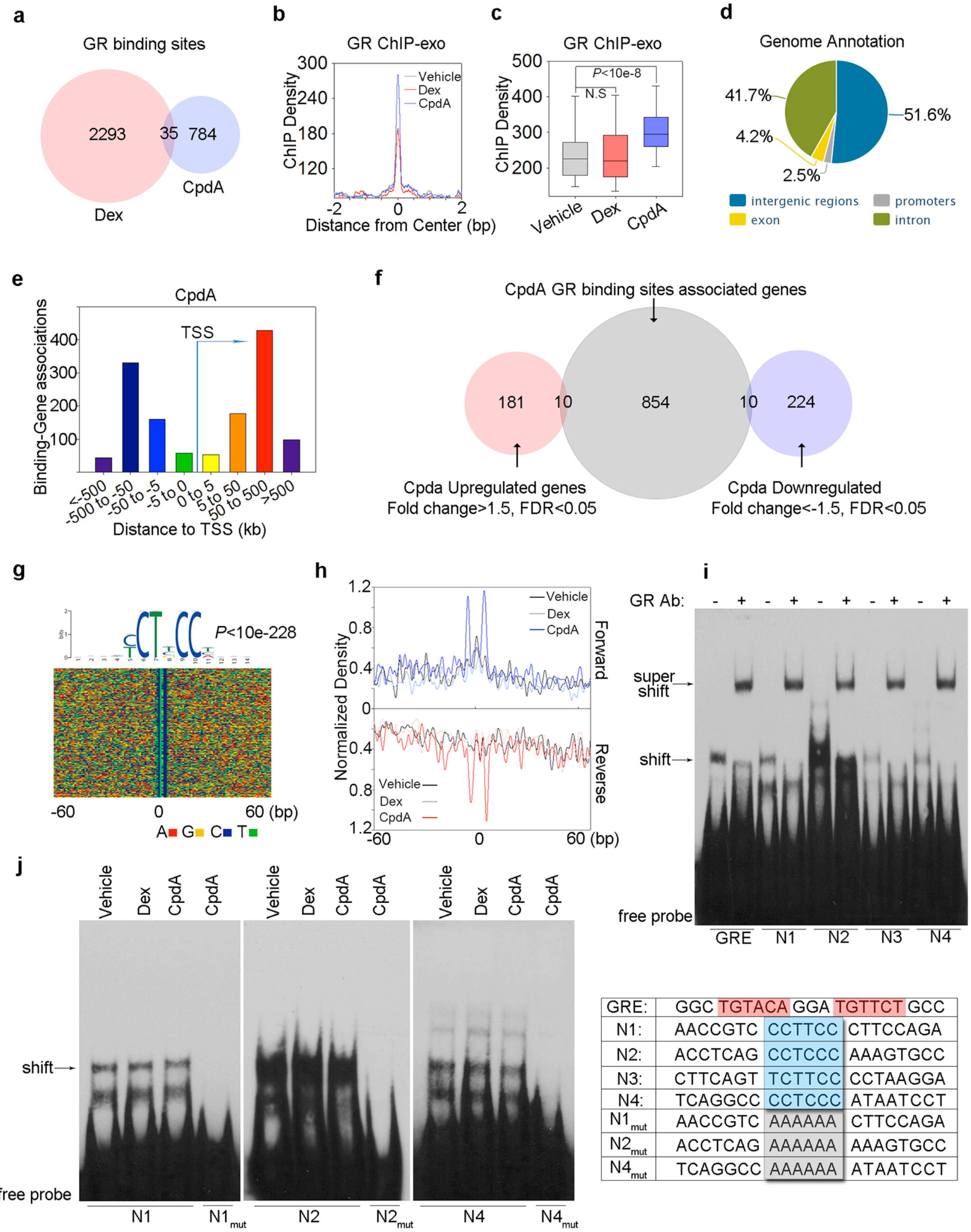

Supplementary Figure 5. Analysis of CpdA-responsive GR binding locations and motifs. (a)

Venn diagram represents overlap between Dex-responsive GR binding locations and CpdA-

responsive GR binding locations. **(b)** An average signal plot and **(c)** a box plot shows ChIP-exo signal densities in CpdA-responsive GR binding locations in cells treated with vehicle, Dex or CpdA. **(d)** Classification of CpdA-responsive GR binding locations based upon annotation. **(e)** Association of CpdA-responsive GR binding with putative regulated genes using the GREAT algorithm. **(f)** Venn diagram compares putative CpdA-liganded GR regulated genes with CpdA-regulated genes determined by RNA-seq. **(g)** CpdA GRE and the sequences are shown. **(h)** Aggregated tag density over CpdA GRE under different treatment conditions is shown on the forward (blue) and reverse (red) strands, separately. **(i, j)** EMSA was performed to validate GR binding to CpdA GRE. Biotin-labeled probes (wild-type and mutants) were incubated with recombinant human GR full-length protein. Anti-GR antibody was also added as indicated at the top. Arrow indicates the position of the shifted and super shifted specific probes. 4 probes (N1-N4) were randomly selected.

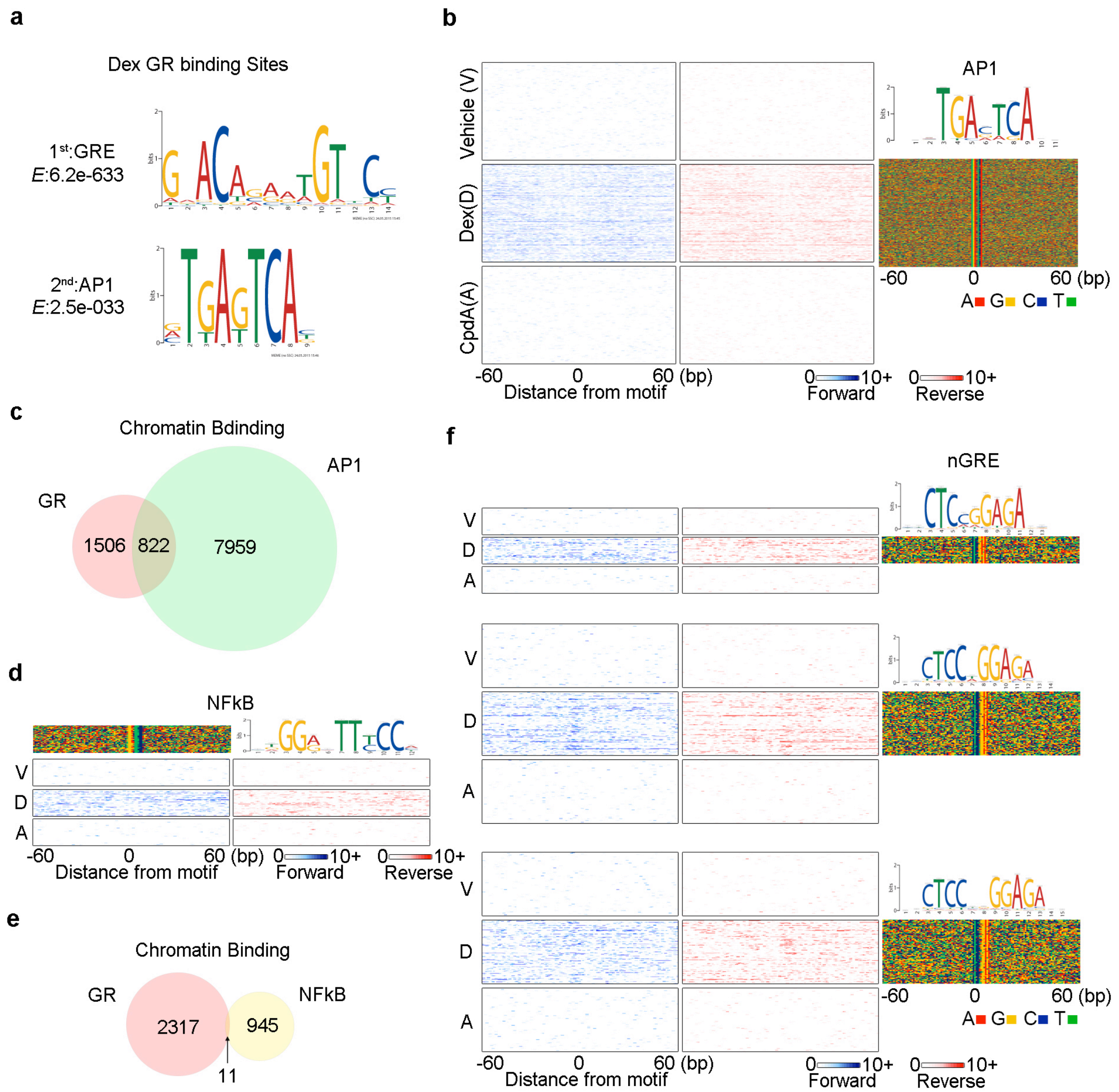

Supplementary Figure 6. *de novo* motif and BPmotif analysis of Dex-responsive GR binding locations. (a) *de novo* motif analysis identified two significantly enriched motifs in Dex-responsive GR binding locations. **(b)** Raw tags distribution over the enriched AP1 motif and under different treatment conditions is shown on the forward (blue) and reverse (red) strands, separately. **(c)** Venn diagram represents overlap between Dex-responsive GR binding locations and AP1 binding locations. **(d)** Raw tags distribution over scanned NF-κB motif and under different treatment conditions is shown on the forward (blue) and reverse (red) strands, separately. **(e)** Venn diagram compares Dex-responsive GR binding locations and NF-κB

binding locations. **(f)** Raw tags distribution over 3 types of scanned nGRE motifs and under different treatment conditions is shown on the forward (blue) and reverse (red) strands, separately.

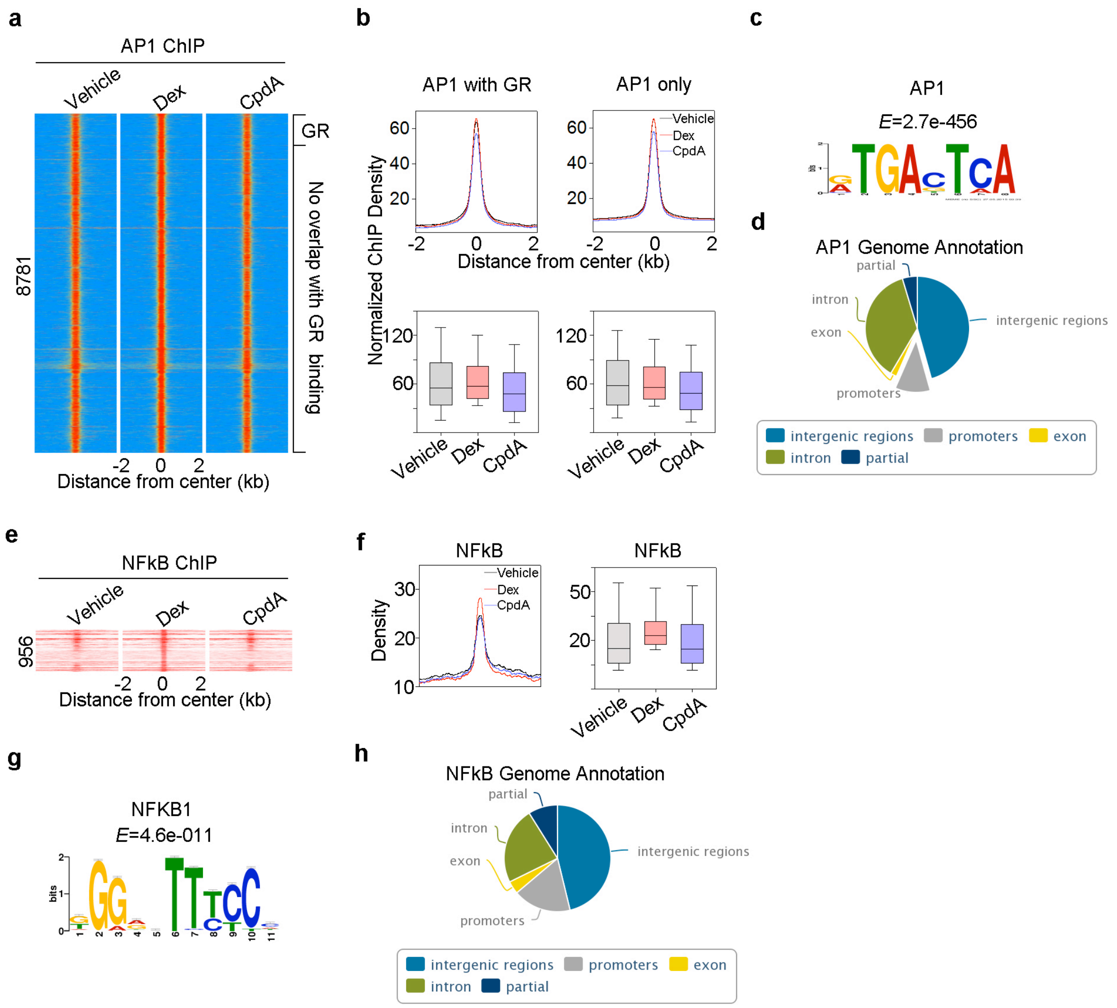

Supplementary Figure 7. ChIP-seq analysis of AP1 and NF-κB in TNBC cells. (a) Heat maps show ChIP-seq signal intensity of AP1 binding in MDA-MB-231 cells treated with vehicle, 100 nM Dex or 10 μM CpdA for 1 h. AP1 binding sites were divided into two classes: those overlapped with GR binding and those not overlapped with GR binding. The number (8,781) indicates total AP1 binding sites. **(b)** Average plots (upper panel) and box plots (lower panel) show ChIP-seq signal densities of AP1 binding in cells treated with vehicle, Dex or CpdA. **(c)** AP1 motif is enriched in AP1 binding sites. **(d)** Classification of AP1 binding locations based upon annotation. **(e)** Heat maps show ChIP-seq signal intensity of NF-κB binding in MDA-MB-

231 cells treated with vehicle, 100 nM Dex or 10 μM CpdA for 1 h. **(f)** Average plots (left panel) and box plots (right panel) show ChIP-seq signal densities of NF-κB binding in cells treated with vehicle, Dex or CpdA. **(g)** NF-κB motif is enriched in NF-κB binding regions. **(h)** Classification of AP1 binding locations based upon annotation.

Supplementary Tables

Supplementary Table 1. Oligo sequences for ChIP-exo sequencing.

AD1+	5'- ACA CTC TTT CCC TAC ACG ACG CTC TTC CGA TCT -3'
AD1-	5'- AGA TCG GAA GAG CGT CGT GTA GGG AAA GAG TGT AG -3'
AD2+	5'- AGA TCG GAA GAG CGG -3'
AD2-	
P2	5'- CGG TCT CGG CAT TCC TGC TGA ACC GCT CTT CCG ATC T -3'
PE1.1	5'- AAT GAT ACG GCG ACC ACC GAG ATC TAC ACT CTT TCC CTA CAC GAC GCT CTT CCG ATC* T -3'
PE2.0	5'- CAA GCA GAA GAC GGC ATA CGA GAT CGG TCT CGG CAT TCC TGC TGA ACC GCT CTT CCG ATC* T -3'

Supplementary Table 2. Primer sequences used in this study.

**ChIP primers**	
BIRC3_ChIP 1+	TCTGTACTCCGCGTACCCT
BIRC3_ChIP 1-	TCAGGGCATCAAATAGCTCAAG
PTPN1_ChIP 1+	GCCAAGTACACGGTCAGTCT
PTPN1_ChIP 1-	CTGGGGCCTTCCTCTTGAAT
NFKBIA_ChIP 1+	GCGAGGTTATTATGAGCTGAGT
NFKBIA_ChIP 1-	GAAGTACAGGGCGTTCCG
NEDD9_ChIP 1+	GCTCTTCTCATGATGGCGAC
NEDD9_ChIP 1-	GGGGTGAGGGTGATTGAGAA
STK4_ChIP 1+	TTGTCTCCCTTGTGTGTTATCT
STK4_ChIP 1-	CAGTAAGGCTACCGTGGTAAA
**RT-PCR primers**	
BIRC3_mRNA 2+	CCAAGTGGTTTCCAAGGTGT
BIRC3_mRNA 2-	TCTCCTGGGCTGTCTGATGT
PTPN1_mRNA 1+	AGACGTCAGTCCCTTTGACC
PTPN1_mRNA 1-	ACTCCTTTGGGCTTCTTCCA
NFKBIA_mRNA1+	GAGCTTTTGGTGTCCTTGGG
NFKBIA_mRNA 1-	ATTACAGGGCTCCTGAGCAT
NEDD9_mRNA 2+	GAAGCATTGGGGGAACCAGT
NEDD9_mRNA 2-	TCACGGGGGTTATCACCTTTTT
STK4_mRNA 1+	CAGGATGGAGACTACGAGTTTCTTA
STK4_mRNA 1-	CTGGTACTTCTGCCGGATCTC

Supplementary Table 3. Probe Sequences used for EMSA assays.

GRE	5'- /5BioTinTEG/CT AGG CTG TAC AGG ATG TTC TGC CTA G -3'
N1	5'- /5BioTinTEG/AG GAA CCG TCC CTT CCC TTC CAG ATC -3'
N2	5'- /5BioTinTEG/TC CAC CTC AGC CTC CCA AAG TGC CAC -3'
N3	5'- /5BioTinTEG/CT CCT TCA GTT CTT CCC CTA AGG ATT -3'
N4	5'- /5BioTinTEG/AA TTC AGG CCC CTC CCA TAA TCC TAT -3'
$N1_{mut}$	5'- /5BioTinTEG/AG GAA CCG TCA AAA AAC TTC CAG ATC -3'
$N2_{mut}$	5'- /5BioTinTEG/TC CAC CTC AGA AAA AAA AAG TGC CAC -3'
$N4_{mut}$	5'- /5BioTinTEG/AA TTC AGG CCA AAA AAA TAA TCC TAT -3'

Supplementary References

1 Rhodes, D. R. *et al.* ONCOMINE: a cancer microarray database and integrated data-mining platform. *Neoplasia* **6**, 1-6 (2004).

2 Curtis, C. *et al.* The genomic and transcriptomic architecture of 2,000 breast tumours reveals novel subgroups. *Nature* **486**, 346-352 (2012).
